# Explicit Content Caching at Mobile Edge Networks with Cross-Layer Sensing

**DOI:** 10.3390/s18040940

**Published:** 2018-03-22

**Authors:** Lingyu Chen, Youxing Su, Wenbin Luo, Xuemin Hong, Jianghong Shi

**Affiliations:** 1 School of Information Science and Technology, Xiamen University, Xiamen 361005, China; 23320161153391@stu.xmu.edu.cn (Y.S.); 23320151154076@stu.xmu.edu.cn (W.L.); xuemin.hong@xmu.edu.cn (X.H.); shijh@xmu.edu.cn (J.S.); 2 Key Lab of Underwater Acoustic Communication and Marine Information Technology, Ministry of Education, Xiamen 361005, China

**Keywords:** edge computing, caching, content delivery, content recommendation, cross-layer design

## Abstract

The deployment density and computational power of small base stations (BSs) are expected to increase significantly in the next generation mobile communication networks. These BSs form the mobile edge network, which is a pervasive and distributed infrastructure that can empower a variety of edge/fog computing applications. This paper proposes a novel edge-computing application called explicit caching, which stores selective contents at BSs and exposes such contents to local users for interactive browsing and download. We formulate the explicit caching problem as a joint content recommendation, caching, and delivery problem, which aims to maximize the expected user quality-of-experience (QoE) with varying degrees of cross-layer sensing capability. Optimal and effective heuristic algorithms are presented to solve the problem. The theoretical performance bounds of the explicit caching system are derived in simplified scenarios. The impacts of cache storage space, BS backhaul capacity, cross-layer information, and user mobility on the system performance are simulated and discussed in realistic scenarios. Results suggest that, compared with conventional implicit caching schemes, explicit caching can better exploit the mobile edge network infrastructure for personalized content dissemination.

## 1. Introduction

The mobile Internet is facing great challenges in coping with ever increasing traffic demand. According to a Cisco white paper, the total amount of global mobile data traffic will reach 49 exabytes per month by 2021 [1]. To cope with the exploding traffic demand, short-range and low-cost small base stations (BSs) will be densely deployed to scale up the capacity of the mobile communication network [2,3,4,5]. These BSs form the mobile edge network, which is a pervasive and distributed infrastructure. It is further envisioned that the BSs will be equipped with extra computing, sensing and caching resources [6], thereby transforming the mobile edge network from a communications-specific infrastructure to a general-purpose edge computing infrastructure [7,8,9]. It is anticipated that such a transform can empower a new wave of location-based and time-sensitive applications [10].

Traffic measurements revealed that the dominant application of the mobile communication network (in terms of the percentage of consumed traffic) has shifted from connection-oriented services, such as phone calls and text messages, to content retrieval services, such as web browsing and video streaming [11]. This fact has inspired the research field of content-centric mobile networks (CCMNs). CCMN recognized that content data/traffic has different characteristics compared with real-time communication data/traffic. For example, content data can be cached in multiple locations before user requests [12], users’ personal preferences for content can be predicted with fair accuracy [13], and the content popularity follows a long-tail distribution [14]. By carefully leveraging these characteristics, various CCMN designs have been proposed to optimize the mobile network for massive content delivery [15]. Among these designs, distributed caching at mobile BSs has emerged as a promising solution that can exploit the pervasive BS infrastructure for effective delivery of massive content data.

BS caching designs can be broadly categorized into two types: implicit caching and explicit caching. Implicit caching means that the cached content is transparent to users, i.e., users are unaware of what contents are cached at the BSs [16]. In this case, users’ requested content is unconstrained and can be any piece of data available on the Internet. A request is answered locally if the target content is cached at the BS (which is called a ‘hit’), otherwise the request is served by a remote server [17,18]. The aim of implicit caching is to cache a subset of contents to maximize the overall possibility of getting a ‘hit’. Implicit caching is a matured technology in the wired Internet [19], but only recently did its application in mobile BSs attract significant research interest. Ref. [20] proposed a content updating method in cache helpers, taking into account the constraint of backhaul capacity and time-varying contents popularity. Ref. [21] conducted a systematic study by exploiting the user mobility in cache-enabled content-centric wireless networks. Ref. [22] investigated the distributed content placement and delivery schemes based on the Manhattan mobility model. Ref. [23,24] exploited the higher-layer knowledge of user mobility and data request preference to pro-actively cache data and provide seamless handover. In summary, most BS caching schemes proposed to date fall into the category of implicit caching. Various aspects such as user demand prediction [25], backhaul constraint [26], and user mobility [27] have been studied in depth. The main advantage of implicit caching lies in decoupling content request and content delivery, therefore the cache facility becomes a communication infrastructure that can provide added value to a broad range of content providers. However, the effectiveness of implicit caching diminishes with a reducing number of users sharing the same cache [28] because the probability of getting a “hit” reduces. Generally speaking, implicit caching would be more effective when deployed closer to the core network at networks that aggregate a large volume of traffic from many users. As cellular networks evolve toward dense deployment of small cells with fewer users per cell [29], the deployment of implicit caching at the mobile edge network is not cost-effective.

To overcome the drawback of implicit caching, we recently proposed the explicit caching scheme [30,31] as an alternative caching paradigm that can better exploit the increasingly dense small cell infrastructure. It relies on the edge computing capability to stored selective content at a BS and exposed the cached content to local users for interactive, localized browsing and download. In this case, users’ requested content is constrained to what is already cached locally. Unlike implicit caching, explicit caching is a different type of edge computing service, which offers joint content recommendation and delivery service to end users. The fundamental advantage of explicit caching is that its utility value (i.e., effectiveness) scales linearly with BS density and cell capacity. In contrast, we note that the effectiveness of the conventional implicit caching scheme decreases at higher BS density. Nevertheless, the explicit caching system also brings unique challenges. The first challenge is to ensure the relevance and attractiveness of cached content. The second challenge is to guarantee that the locally-generated traffic does not degrade the incumbent traffic coming to/from the core network. The third challenge is to guarantee the reliability and timeliness of local content delivery to sustain a satisfactory quality-of-experience (QoE). These three challenges urge a holistic cross-layer design that can jointly optimize content recommendation and content delivery in the explicit caching system.

To the best of our knowledge, research about explicit BS caching systems is still in its infancy. In a conference version of this work [31], we made an initial attempt to propose a cross-layer design architecture and present some initial results. This article extends [31] to a systematic study. Our main contributions are summarized below:

- First, a comprehensive and extended system model is presented, taking into account various aspects such as content property, user interest, user mobility, BS cache space, BS backhaul capacity, and cross-layer sensing capability.
- Second, theoretical performance bounds are derived to estimate the utility of the explicit caching system in a simple yet representative scenario.
- Third, optimal algorithms are given solving the explicit caching problem. Low-complexity heuristic algorithms are also proposed and showed to yield marginal performance degradation compared with the optimal ones.
- The impacts of various parameters on the system performance are systematically evaluated and discussed via simulations. Key design guidelines of the explicit caching system are summarized for future system developers.

The remainder of this paper is organized as follows. Section 2 introduces the system model. Section 3 formulates the content placement problem with cross-layer considerations. Section 4 presents the optimal and heuristic content caching algorithms. Section 5 analyzes the theoretical performance bounds of the system with a simplified model, followed by simulation-based performance evaluation with realistic models in Section 6. Finally, Section 7 concludes the paper.

## 2. System Model

### 2.1. Cellular Network Model and Description of the Explicit Caching Service

**Figure 1:**
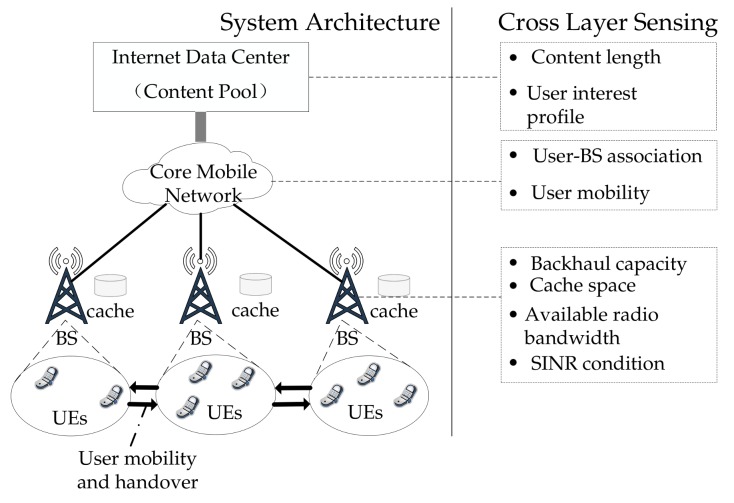
Illustration of the system model.

As illustrated in Figure 1, we consider a mobile edge network with multiple BSs and users, where each user is associated with only one BS. Each BS runs independently to select and retrieve content from a remote Internet Data Center (IDC) and stores the content at a local cache. The explicit caching service allows a user to browse the cached content of the associated BS and download/view the content according to their personal interest. Essentially, the explicit caching service resembles an FTP service in a local network. The backhaul link connecting each BS and the IDC can either be fixed or wireless connections, while each link is characterized by a limited capacity. Time is slotted into periods of $T_{s}$ seconds. At the beginning of each slot, a BS makes a decision on how to update its cached content. The decision should simultaneously address multiple concerns including the content’s attractiveness to local users (i.e., the recommendation problem), the constraints of cache space and backhaul capacity (i.e., the caching problem), and the constraint of radio access capacity (i.e., the content delivery problem). It is assumed that the BSs run distributed and independent algorithms of explicit caching, hence our study can focus on a single typical BS.

From the mobile operators’ perspective, it is very important for the explicit caching service to coexist peacefully with other existing cellular services. Following the proposal in [30], we assume that the explicit caching service is offered as a “secondary service”, which means that it has a lower priority to utilize the radio resource at the BS than conventional services (which are primary services). After radio resource contention, we assume that only a portion of the system bandwidth is left for the explicit caching service. The available bandwidth is assumed to be stable for the time frame of interest and is denoted as *W*. Apart from the radio bandwidth, other physical resource constraints include the cache storage space *S* (bits) and backhaul capacity δ (bits). The backhaul capacity caps the maximum number of bits updated between two consecutive time slots.

Users in the cellular network are assumed to be mobile. Let K denote the set of potential users that may be served by the BS over the long term. Users are assumed to be mobile, which means that they move in and out of coverage from time to time. User mobility is captured by a two-state Markov chain, whose transition probability matrix **$P_{0}$** is given by

(1) $$P_{0}=\left[\begin{array}{cc} 1‒P_{a} & P_{a}\\ P_{b} & 1‒P_{b} \end{array}\right],$$

where $P_{a}$ and $P_{b}$ denote the probability for a user to move within and out of coverage, respectively. At a time instance, only a subset of users are connected to the BS. The set of connected users is denoted as U. The sizes of K and U are denoted as *K* and *U*, which represent the number of all users and the number of connected users in a cell, respectively.

### 2.2. Content Models

It is assumed that a large content pool F with *F* pieces of files are stored at the remote IDC. The length of the *f*-th (f=1,…,F) file is denoted by $L_{f}$. Based on users’ preference, a recommender system computes a numerical value $I_{k,f}$ to indicate the interest of the *k*-th (k=1,2,…,K) user on the *f*-th file. The user interest model is captured by a K×F matrix **I**, whose entries are given by $I_{k,f}$. For an arbitrary pair of *k* and *f*, we assume that $I_{k,f}$ follows independent and identical distributions (i.i.d.). Similarly, $L_{f}$ also follows i.i.d. distributions. Different models can be used to describe the statistical properties of $I_{k,f}$ and $L_{f}$. In our paper, we further distinguish two content models: a simple model and a realistic model. The former is used to facilitate theoretical analysis, while the latter is used for simulations:

- Simple content model: The user interest parameter $I_{k,f}$ is assumed to be a binary random variable ∈{0,1} with mean ε. The file length $L_{f}$ follows a uniform distribution in [$l_{min},l_{max}$].
- Realistic content model: According to reported measurements, we assume that $I_{k,f}$ is jointly characterized by two distributions. First, the average popularity of different files (i.e., $\sum_{k=1}^{K}I_{k,f}/K$) follows a Zipf distribution with parameter β [14]; second, the interests of different users on a particular file follow a Gaussian distribution [32]. The file length $L_{f}$ follows a log-normal distribution [33].

### 2.3. Transmission Model

Let $D_{k,f}$ denote the time required for the *k*-th user to download the *f*-th file. We have

(2) $$D_{k,f}=L_{f}/C_{k},$$

where $L_{f}$ is the file length and $C_{k}$ is the instantaneous downlink capacity of the *k*-th user. The file length $L_{f}$ is a random variable following a log-normal distribution [33], while the capacity is given by

(3) $$C_{k}=\frac{W}{U}\log_{2}\left(1+\frac{P_{t}d_{k}^{‒\alpha }g_{m}}{W\sigma^{2}/U+I_{k}}\right),$$

where $P_{t}$ is the transmit power per user, $\sigma^{2}$ is the constant noise power of each user, and $I_{k}$ denotes the accumulated interference perceived by the *k*-th user. In practice, $I_{k}$ is dominated by the accumulated inter-cell interference from other co-channel cells. According to the literature of interference modeling and capacity analysis in large scale cellular networks, the accumulated inter-cell interference can be approximately treated as Gaussian noise when evaluating the link capacity [34]. This widely used approximation is also adopted in our paper for simplicity. $d_{k}$ is the distance between BS and the *k*-th user, α is the path-loss exponent, and $g_{m}$ is a fast fading coefficient following an exponential distribution of unit mean (i.e., Rayleigh fading). It is assumed that the available bandwidth *W* is equally shared among users. Let $\gamma_{k}$ denote the *k*-th user’s SINR (Signal to Interference plus Noise Ratio) averaged over small scale fading, we can then rewrite Equation (3) as

(4) $$C_{k}=\frac{W}{U}\log_{2}\left(1+\gamma_{k}g_{m}\right).$$

### 2.4. User QoE Model

The ultimate goal of the explicit caching system is to maximize the users’ QoE of content consumption. Two factors are considered to have critical impacts on users’ QoE. The first factor is the attractiveness of content, which is captured by the user interest parameter $I_{k,f}$. The second factor is the time/delay $D_{k,f}$ for downloading a content file. Each user is assumed to have the same delay tolerance, which is captured by a time threshold $T_{0}$. The QoE for the *k*-th user to download and view the *f*-th content is then defined as

(5) $$Q_{k,f}=I_{k,f}Z_{k,f},$$

where $Z_{k,f}$ is defined as

(6) $$Z_{k,f}=\left\{\begin{array}{cc} \xi , & D_{k,f}\leq T_{0},\\ 0, & D_{k,f}>T_{0}. \end{array}\right.\;$$

In Equation (6), ξ is a scaling constant with a positive real value. The above definition means that, if a requested file can be delivered in time, the QoE has a positive value proportional to the user’ interest on the file; otherwise, the QoE is zero.

Finally, for the convenience of readers, Table 1 summarizes the major parameters and their physical meanings in our system model.

## 3. Problem Formulation with Cross-Layer Consideration

### 3.1. General Problem Formulation

The aim of this paper is to find a content placement policy that can maximize the expected QoE of users over a long time. The update of content is subject to constraints on the cache space *S* and backhaul capacity δ. The cache space constraint is memoryless in a sense that it does not impose connections between two consecutive time slots.

**Figure 2:**
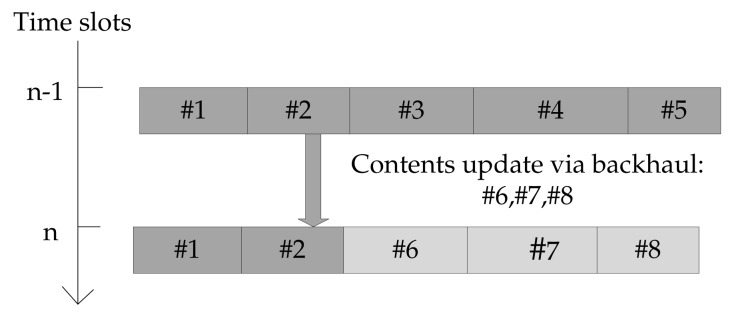
Illustration of content update between two successive time slots, (#6,#7,#8) represent the contents updated via the backhaul network.

However, as illustrated in Figure 2, the backhaul capacity constraint introduce historical dependencies into the system and desires joint considerations over multiple time slots. As a result, our paper will address the general problem of content placement over multiple time slots, noting that the single-slot problem is a special case of the general problem. We further note that the multi-slot problem implies that the information of critical system status (e.g., user association) is available for all the slots under consideration. In practice, this can be achieved by forward prediction (assuming ideal prediction accuracy).

Let *n* denote the index of time slot. Further define a binary variable $x_{f}\left(n\right)$∈{0,1}, which denotes whether the *f*-th file is cached (taking value 1) or not (taking value 0) during the *n*-th time slot. The required backhaul traffic for a transition from the (n‒1)-th slot to the *n*-th slot can be evaluated as

(7) $$\sum_{f=1}^{F}L_{f}\left\{x_{f}\left(n\right)‒x_{f}\left(n‒1\right)\right\}x_{f}\left(n\right).$$

Let $V_{f}\left(n\right)$ denote the expected QoE per user for the *f*-th file during the *n*-th time slot. We have

(8) $$\begin{array}{cc} V_{f}\left(n\right) & =E\left\{\frac{1}{U(n)}\sum_{k\in U}Q_{k,f}\left(n\right)\right\}\\ & =\frac{1}{U(n)}\sum_{k\in U}I_{k,f}E\left\{Z_{k,f}\left(n\right)\right\}, \end{array}$$

where **E**{·} means taking expectation. Here, the expectation is taken over the fast channel fading coefficient. The users’ interest profile is assumed to be consistent over multiple time slots. However, the number of users connected to the BS, as well as the user-BS distances, change from slot to slot. Therefore, U(n), $C_{k}\left(n\right)$ and $Z_{k,f}\left(n\right)$ should all be treated as time-varying functions. According to Equation (8), the value of a file/content is measured by the sum QoE of all the active users of a BS. A user is active when he is currently associated with the BS and runs the explicit caching service. Fairness among active users is embedded in such a QoE measure because each active user essentially “votes” to cache the most interested and deliverable contents. In practice, the set of active users changes dynamically and an inactive user may become active. In this case, our caching policy can dynamically update the cached content via backhaul to ensure user fairness among the new set of active users.

The problem of joint content placement and delivery, which aims to maximize the expected user QoE over a time span of *N* slots, can then be formulated as

(9) $$\begin{array}{cc} \left(P_{1}\right) & \;\max_{x}\sum_{n=1}^{N}\sum_{f=1}^{F}V_{f}\left(n\right)x_{f}\left(n\right)\\ & \;st.\sum_{f=1}^{F}L_{f}x_{f}\left(n\right)\leq S,\forall n\\ & \;\;\sum_{f=1}^{F}L_{f}\left(x_{f}\left(n\right)‒x_{f}\left(n‒1\right)\right)x_{f}\left(n\right)\leq \delta ,\forall n\\ & \;\;x_{f}\left(n\right)\in \left\{0,1\right\}. \end{array}$$

The optimization problem in ($P_{1}$) formulates the explicit caching problem as a joint content recommendation, caching, and delivery problem. The aspect of content recommendation is reflected by the fact that the optimization decision is to select a subset of content files that tends to maximize the total user interest; the aspect of content caching is reflected by the constraints of storage space *S* and backhaul capacity δ; the aspect of content delivery is reflected by modelling the utility function to be dependent on the content delivery delay, which is a major performance metric for content delivery systems. Our problem formulation unifies all the key parameters that appear in the processes of content recommendation, caching, and delivery.

Two difficulties arise in solving this optimization problem. First, the expectation operation appeared within the utility function should be analytically evaluated for tractability; second, the problem appears to be a nonlinear 0–1 integer programming problem, which is non-deterministic polynomial-time hard (NP-hard) and difficult to solve directly. In what follows, we will first deal with the first difficulty by proposing an analytical approximation for the utility function.

### 3.2. Analytical Approximation of the Utility Function

Small cell users typically enjoy a high SINR. Considering the high SINR regime, we can approximate Equation (4) as

(10) $$C_{k}=\frac{W}{U\mathrm{lg}2}\mathrm{lg}\gamma_{k}+\frac{W}{U\mathrm{lg}2}\mathrm{lg}g_{m}.$$

Let $q=‒C_{k}$, we can show that *q* follows a generalized extreme value (GEV) distribution with three parameters given by $\mu =‒W\mathrm{lg}\gamma_{k}/U\mathrm{lg}2$, σ=W/Ulg2, ξ=0. The CDF of *q* is

(11) $$F_{q}\left(x\right)=\exp\left\{‒\exp\left[‒Ux\mathrm{lg}2/W‒\mathrm{lg}\gamma_{k}\right]\right\}.$$

Given Equation (11), the expectation of $Z_{k,f}\left(n\right)$ defined in Equation (6) can be evaluated as

(12) $$\begin{array}{cc} E\{Z_{k,f}\left(n\right)\} & =\xi P(L_{f}/C_{k}\left(n\right)\leq T_{0})\\ & =\xi P(L_{f}/T_{0}\leq C_{k}\left(n\right))\\ & =\xi P(‒C_{k}\left(n\right)\leq ‒L_{f}/T_{0})\\ & =\xi F_{q}\left(‒L_{f}/T_{0}\right)\\ & =\xi \exp\left\{‒\exp\left[U\left(n\right)L_{f}\mathrm{lg}2/T_{0}W‒\mathrm{lg}\left[\gamma_{k}\left(n\right)\right]\right]\right\}. \end{array}$$

Substituting Equation (12) into Equation (8), the utility function can be evaluated in closed-form.

### 3.3. Problem Variations with Cross-Layer Consideration

The original optimization problem ($P_{1}$) implies that the optimizer has full cross-layer information, which not only includes higher layer (i.e., application layer) information such as the content popularity profile, content length profile, and user interest profile, but also includes lower layer information such as user association (information at the medium access control (MAC) layer) and user channel path-loss (information at the physical layer). It is worth noting that the cross-layer analytical framework proposed in this paper is different from the conventional frameworks of cross-layer analysis [35,36]. As shown in Figure 1, both ours and the conventional frameworks consider multiple protocol stacks from the top application layer to the bottom physical layer. However, conventional frameworks only address the data-level of the application layer, while our framework goes one step further to address the semantic-level of the application layer by bringing content recommendation into the design space. As a result, our problem formulation resembles a classic recommendation problem [37], and differs from the network utility maximization problem usually appeared in conventional cross-layer literature [38]. Unification of these two types of frameworks is also a promising area for future research. In reality, however, it is difficult or even impractical to obtain the exact lower layer information. Depending on the capability of cross-layer sensing and the availability of lower layer information, we can further distinguish the following three cases.

Case 1: In this case, both the user association (i.e., which users are currently served by the BS) and user SINR are known. This is the ideal case with full cross-layer information. The utility function is then given by

(13) $$V_{f}^{c1}\left(n\right)=\frac{1}{U\left(n\right)}\sum_{k\in U}I_{k,f}\xi \exp\left\{‒\exp\left[U\left(n\right)L_{f}\mathrm{lg}2/T_{0}W‒\mathrm{lg}\left[\gamma_{k}\left(n\right)\right]\right]\right\}.$$

Case 2: In this case, the user association is known, but the user SINR $\gamma_{k}$ is unknown. However, we assume that the average SINR $\bar{\gamma }$ of all users is known. It follows that the $\gamma_{k}$ in Equation (12) should be replaced by $\bar{\gamma }$. The utility function should be changed correspondingly into

(14) $$V_{f}^{c2}\left(n\right)=\frac{1}{U\left(n\right)}\sum_{k\in U}I_{k,f}\xi \exp\left\{‒\exp\left[U\left(n\right)L_{f}\mathrm{lg}2/T_{0}W‒\mathrm{lg}\left(\bar{\gamma }\right)\right]\right\}.$$

Case 3: In this case, both the user association and user SINR are unknown. However, the number of users and the average SINR is known. The utility function should now consider all users and is given by

(15) $$V_{f}^{c3}=\frac{1}{K}\sum_{k=1}^{K}I_{k,f}\xi \exp\left\{‒\exp\left[KL_{f}\mathrm{lg}2/T_{0}W‒\mathrm{lg}\left(\bar{\gamma }\right)\right]\right\}.$$

We can see that Case 1 has a ‘true’ utility function and yields the global optimal performance. On the contrary, Cases 2 and 3 have biased utility functions, and hence their solutions may be distorted. If all the three cases are evaluated according to the same ‘true’ utility function, we can expect to see some performance gaps between Case 1 and Case 2 /Case 3. Such performance gaps will indicate the usefulness of different sources of cross-layer information. The optimization problems of these three cases have the same structure as the original problem of $\left(P_{1}\right)$, which appears to be a difficult nonlinear combinatorial optimization problem. In the following section, we will introduce the algorithms to solve $\left(P_{1}\right)$.

## 4. Optimal and Heuristic Content Placement Algorithms

### 4.1. Problem Linearization and Optimal Algorithm

The nonlinearity of optimization problem in ($P_{1}$) comes from the second constraint, which is imposed by limited backhaul capacity. Let us introduce a new variable $y_{f}\left(n\right)=x_{f}\left(n‒1\right)x_{f}\left(n\right)$. Based on this definition, we can write

(16) $$\sum_{f=1}^{F}L_{f}x_{f}\left(n\right)‒\sum_{f=1}^{F}L_{f}y_{f}\left(n\right)\leq \delta .$$

According to the very definition of $y_{f}\left(n\right)$, we introduce two additional linear constraints as follows:

(17) $$\begin{array}{cc} y_{f}\left(n\right) & \leq x_{f}\left(n‒1\right),\\ y_{f}\left(n\right) & \leq x_{f}\left(n\right). \end{array}$$

Substituting Equations (16) and (17) into ($P_{1}$), the original multi-slot content placement problem can be transformed into a linear 0–1 integer programming problem given by

(18) $$\begin{array}{cc} \left(P_{2}\right) & \;\max_{x}\sum_{n=1}^{N}\sum_{f=1}^{F}V_{f}\left(n\right)x_{f}\left(n\right)\\ & \;st.\sum_{f=1}^{F}L_{f}x_{f}\left(n\right)\leq S,\forall n\\ & \;\;\sum_{f=1}^{F}L_{f}x_{f}\left(n\right)‒\sum_{f=1}^{F}L_{f}y_{f}\left(n\right)\leq \delta ,\forall n\\ & \;\;y_{f}\left(n\right)\leq x_{f}\left(n‒1\right)\\ & \;\;y_{f}\left(n\right)\leq x_{f}\left(n\right)\\ & \;\;x_{f}\left(n\right)\in \left\{0,1\right\}\\ & \;\;y_{f}\left(n\right)\in \left\{0,1\right\}. \end{array}$$

We note that in ($P_{2}$), $x_{f}$(n) and $y_{f}$(n) are all treated as decision variables in the optimization problem. We will subsequently prove that ($P_{2}$) is equivalent to ($P_{1}$).

**Proof.**  The key is to demonstrate that $y_{f}$(n) = $x_{f}\left(n‒1\right)x_{f}\left(n\right)$ will always hold for the optimum solutions of ($P_{2}$), so that the newly-introduced variable in ($P_{2}$) does not change the decision space of ($P_{1}$). The proof starts by considering two complementary cases.

In the first case, either $x_{f}$(n) or $x_{f}\left(n‒1\right)$ is zero. According to Equation (17), we have $y_{f}\left(n\right)=0$. In this case, $y_{f}\left(n\right)=x_{f}\left(n‒1\right)x_{f}\left(n\right)$ holds true, and hence ($P_{2}$) is equivalent to ($P_{1}$).

In the second case, both $x_{f}\left(n‒1\right)$ and $x_{f}\left(n\right)$ are equal to 1. We have $y_{f}=1$ in ($P_{1}$), but $y_{f}\left(n\right)$ can be either 0 or 1 in ($P_{2}$) according to Equation (18). We can then write the second constraint in ($P_{2}$) as

(19) $$\sum_{f=1}^{F}L_{f}x_{f}\left(n\right)\leq \delta +\sum_{f=1}^{F}L_{f}y_{f}\left(n\right).$$

However, because $y_{f}\left(n\right)$ is an optimization variable in ($P_{2}$), we can see that $y_{f}\left(n\right)=1$ is always a better solution than $y_{f}\left(n\right)=0$ in this case. Consequently, the optimization process will ensure $y_{f}\left(n\right)=1$, which means $y_{f}\left(n\right)=x_{f}\left(n‒1\right)x_{f}\left(n\right)$ holds true for the optimal solution of ($P_{2}$), and hence ($P_{2}$) is equivalent to ($P_{1}$). ☐

Because ($P_{2}$) is linear, the optimal algorithm to solve the problem is the classic branch and bound algorithm [39,40,41]. However, due to the combinatorial nature of the problem ($P_{2}$), the complexity of the optimal algorithm scales exponentially with the problem size (i.e., the number of files). In the special case of N=1, the original multi-slot content placement problem is simplified to a single-slot content placement problem. It is easy to show that the single-slot problem is a classic two-dimensional 0–1 knapsack problem, which can be solved by dynamic programming with a pseudo-polynomial time complexity [42]. In practice, however, the pseudo-polynomial complexity can still become exponentially complex in the worse case. In the context of edge computing, it is desirable to have low-complexity algorithms with a strict polynomial time complexity. To this end, low-complexity heuristic algorithms will be introduced subsequently.

### 4.2. Heuristic Algorithms

#### 4.2.1. Heuristic Algorithm for the Single Time Slot Problem

We first present a heuristic algorithm for the single time-slot problem (i.e., N=1). This algorithm will serve as the basis for the heuristic algorithm used for a multiple time slot problem. The proposed algorithm is based on a simple greedy heuristic: cache files with the largest file value per unit length until the cache space or the backhaul capacity exceed the constraints. The core of this algorithm is a sorting operation, hence the algorithm has a polynomial complexity given by O(FlogF).

#### 4.2.2. Heuristic Algorithm for the Multiple Time Slot Problem

A major drawback of the single time slot algorithm is that the “ping-pong” phenomenon may occur, which means that a file is cached in slot *n*, deleted in slot n+1, and cached again in slot n+2. This phenomenon will impose an unnecessary burden on the backhaul and reduce the overall caching performance. Compared with the single time slot optimization, a major advantage of multi-slot optimization is to eliminate the ping-pong phenomenon. This inspires us to introduce a heuristic algorithm. The rationale is to first perform independent single slot optimization for three consecutive time slots. If the ping-pong phenomenon is observed (i.e., find a file whose decision variable is 1-0-1 for three consecutive slots), eliminate the phenomenon by enforcing the decision variable to 1-1-1. Our heuristic algorithm operates on three time slots because at least three time slots are required to detect the ping-pong phenomenon. The pseudo-code of this algorithm is shown in Algorithm 1. Similar to the single-slot algorithm, Algorithm 1 also has a polynomial complexity given by O(FlogF).

**Algorithm 1** Heuristic algorithm for the multi-slot problem
**Input:** $x^{\prime }$, *x*, *v*, *w*, σ, *n*, *I*
**Output:** $x_{op}$
1:**Initialization:** Let x’ denotes decision variables of the last slot, *x* is the decision variables of the current slot, *v* represents the file value per unit length, *w* and σ indicates the cache space and update threshold, *n* is the number of files, *I* indicates maximum iteration number and *i* indicates the iteration index2:Step1: Obtain decision variables of three consecutive slots with the optimal algorithm in single-slot senario and the overall cache value *V*3:Step2: Find a file set Φ whose decision variable is 1-0-1 for three consecutive slots4:**if** Φ=∅ **then**5:    Go to Step66:Step3:7:**if** I<i **then**8:    Go to Step69:Step4: Randomly choose a file f∈Φ and change its decision variable to 1-1-1, then use heuristic algorithm for the single time slot problem to recalculate the decision variables for the remaining files in the last two slots and overall value $V^{\prime }$10:Step5:11:**if** $V\leq V^{\prime }$ **then**12:    Update $x_{op}=x$ and $V=V^{\prime }$13:**else**14:    Change decision variables back to their original ones and return to Step3.15:Step6: **return** optimal decision variables $x_{op}$


## 5. Theoretical Performance Bounds with Simplified Model

The value of an explicit caching system can be evaluated by the expected user QoE. So far, although algorithms to maximize the user QoE have been obtained, we still lack a clear analytical insight into how the user QoE is related to various system parameters. This section aims to derive some theoretical performance bounds of the explicit caching system. For analytical tractability, we will apply the simple content model introduced in Section 2.2. Moreover, the following simplifying assumptions are further made in this section.

- The system performance is evaluated according to the utility function presented by Equation (14), which corresponds to Case 2. We recall that this case assumes that the user association and average user SINR is known. In this section, we will treat the number of users and the average SINR as fixed value parameters and denote them as *U* and $\bar{\gamma }$, respectively. Although this section focuses on Case 2 only, we will show the latter in Section 6 that the performance of Case 2 serves as a very good predictor (almost identical) to the ideal performance in Case 1.
- The cache space constraint *S* is replaced by a file number constraint *M*, which limits the maximum number of files that can be cached. This simplification is reasonable because the value of the cached content tends to concentrate on a subset of high-value files. In other words, a fixed number of high-value files will capture most of the value of the entire cached file set. This simplification implies that our analysis in this section should be interpreted as an approximation.

The above assumptions yield a new optimization problem given by

(20) $$\begin{array}{cc} \left(P_{3}\right) & \;Q=\max_{x}\sum_{n=1}^{N}\sum_{f=1}^{F}V_{f}^{c2}\left(n\right)x_{f}\left(n\right)\\ & \;st.\sum_{f=1}^{F}x_{f}\left(n\right)=M,\forall n\\ & \;\;\sum_{f=1}^{F}L_{f}x_{f}\left(n\right)‒\sum_{f=1}^{F}L_{f}y_{f}\left(n\right)\leq \delta ,\forall n\\ & \;\;y_{f}\left(n\right)\leq x_{f}\left(n‒1\right)\\ & \;\;y_{f}\left(n\right)\leq x_{f}\left(n\right)\\ & \;\;x_{f}\left(n\right)\in \left\{0,1\right\}\\ & \;\;y_{f}\left(n\right)\in \left\{0,1\right\}. \end{array}$$

This new problem, which is analytically tractable, can serve as an approximation to the original problem.

### 5.1. CDF of the Utility Function

The first step is to evaluate the CDF of the utility function $V_{f}^{c2}\left(n\right)$, which can be written as the product of two independent random variables

(21) $$V_{f}^{c2}\left(n\right)=XY,$$

where

(22) $$\begin{array}{cc} X & =c\exp[‒\exp\left(aL_{f}+b\right)],\\ Y & =\sum_{k\in U}I_{k,f}. \end{array}$$

Here, we have $c=\xi /U\left(n\right)$, $a=U\left(n\right)\mathrm{lg}2/T_{0}W$, and $b=‒\mathrm{lg}\bar{\gamma }$.

Let us first focus on the CDF of the random variable *X*. Given uniform file size distribution, the CDF of the random file length $L_{f}$ is given by

(23) $$F_{L_{f}}\left(l\right)=\left\{\begin{array}{cc} 0, & l<l_{min},\\ \frac{l‒l_{min}}{l_{max}‒l_{min}}, & l_{min}\leq l\leq l_{max},\\ 1, & l>l_{max}. \end{array}\right.\;$$

Because *X* is a function of $L_{f}$, it follows that CDF of *X* can be derived as

(24) $$\begin{array}{cc} F_{X}\left(x\right) & =P(X\leq x)\\ & =P\left(\exp\left[‒\exp\left(aL_{f}+b\right)\right]\leq \frac{x}{c}\right)\\ & =P\left(\exp\left[aL_{f}+b\right]\geq \ln\left(\frac{c}{x}\right)\right)\\ & =P\left(L_{f}\geq \frac{1}{a}\ln\left(\ln(\frac{c}{x})\right)‒\frac{b}{a}\right)\\ & =1‒P\left(L_{f}\leq \frac{1}{a}\ln\left(\ln(\frac{c}{x})\right)‒\frac{b}{a}\right)\\ & =1‒F_{L_{f}}\left(\frac{1}{a}\ln\left(\ln(\frac{c}{x})\right)‒\frac{b}{a}\right)\\ & =\left\{\begin{array}{cc} 0, & x<x_{min}\\ 1‒\frac{\ln\left(\ln(\frac{c}{x})\right)+al_{min}+b}{a(l_{max}‒l_{min})}, & x_{min}\leq x\leq x_{max}\\ 1, & x>x_{max}, \end{array}\right.\; \end{array}$$

where $x_{min}$ = $c\exp[‒\exp\left(al_{max}+b\right)]$, $x_{max}$ = $c\exp[‒\exp\left(al_{min}+b\right)]$.

Let us now consider the random variable *Y*. For a total of *U* users linked to the BS, the probability for an arbitrary user to be interested in the *f*-th file (i.e., $I_{k,f}=1$) is

(25) $$P=\frac{A_{f}}{U},$$

where $A_{f}$ is the aggregated user interest of the *f*-th file. Because the user interest profiles are independent, random variable *Y* obeys a binomial distribution given by Y∼Bi(U,P).

The utility function $Z=V_{2}^{c2}$ is a product of random variables *X* and *Y*, hence its CDF can be derived as

(26) $$F_{Z}\left(z\right)=\sum_{y_{i}>0}p_{i}F_{X}\left(\frac{z}{y_{i}}\right)+\sum_{y_{i}<0}p_{i}\left(1‒F_{X}\left(\frac{z}{y_{i}}\right)\right)+p_{y_{i}=0}F_{X}\left(\frac{z}{0}\right).$$

Because random variable *Y* is nonnegative, we can omit the situation when $y_{i}<0$. It follows that Equation (26) can be further refined as

(27) $$\begin{array}{cc} F_{Z}\left(z\right) & =\sum_{y_{i}>0}p_{i}F_{X}\left(\frac{z}{y_{i}}\right)+p_{y_{i}=0}F_{X}\left(\frac{z}{0}\right)\\ & =\sum_{i=1}^{U}\left(\begin{array}{c} U\\ i \end{array}\right)P^{i}{\left(1‒P\right)}^{U‒i}F_{X}\left(\frac{z}{i}\right)+\left(\begin{array}{c} U\\ 0 \end{array}\right)P^{0}{\left(1‒P\right)}^{U}. \end{array}$$

### 5.2. Bounds of Average User QoE Performance

Once the CDF of the utility function is known, we can move on to analyze the outcome *Q* (i.e., optimized average user QoE) of the optimization problem defined (P3). We distinguish two extreme cases that correspond to the lower bound and upper bound of the optimized QoE performance.

In case of the lower bound, the optimization problem (P3) is solved by a random caching policy, which randomly choose *M* files from the content pool. Such a random policy is optimal in two situations: during initialization or when the backhaul capacity is zero. This is because both situations should assume that all users are equally probable to be served by the BS over the long term, and hence each piece of content would have the same value over the long term. It follows that the expected outcome can be evaluated as

(28) $$V_{low}=M\tilde{V},$$

where $\tilde{V}$ represents the expected value of a randomly chosen file, which can be calculated as

(29) $$\tilde{V}=\int zf_{Z}\left(z\right)dz,$$

where $f_{Z}\left(z\right)$ (i.e., PDF) is the first-order differentiation of $F_{Z}\left(z\right)$.

In case of the upper bound, the optimization problem is solved by a greedy policy, which always picks the most valuable *M* files. The policy is feasible when the backhaul capacity is unlimited (or large enough to support any update). According to the results of order statistics [43,44,45], given *F* i.i.d., random variables with CDF denoted by F(x), the CDF of the *r*-th largest random variable is given by

(30) $$G_{\left(r\right)}\left(x\right)=\sum_{i=r}^{F}\left(\begin{array}{c} F\\ i \end{array}\right)F^{i}\left(x\right){\left[1‒F\left(x\right)\right]}^{F‒i}.$$

The PDF of the *r*-th order statistics is

(31) $$g_{\left(r\right)}\left(x\right)=\frac{dG_{\left(r\right)}\left(x\right)}{dx}.$$

The expectation of the *r*-th order random variable is

(32) $$E\left\{X_{r}\right\}=\int_{x_{min}}^{x_{max}}xg_{\left(r\right)}\left(x\right)dx,$$

where $g_{\left(r\right)}\left(x\right)$ is the corresponding probability density function (PDF) of $G_{\left(r\right)}\left(x\right)$. The expectation of the most valuable *M* files can be calculated as

(33) $$\begin{array}{cc} V_{up} & =E\{X_{F‒M+1}+X_{F‒M+2}+\ldots +X_{F}\}\\ & =E\left\{X_{F‒M+1}\right\}+E\left\{X_{F‒M+2}\right\}+\ldots +E\left\{X_{F}\right\}\\ & =\int_{x_{min}}^{x_{max}}x\left[g_{\left(F‒M+1\right)}\left(x\right)+g_{\left(F‒M+2\right)}\left(x\right)+\ldots +g_{\left(F\right)}\left(x\right)\right]dx. \end{array}$$

### 5.3. Validation of the Theoretical Bounds

In this subsection, we carry out Monte Carlo simulations to validate the derived theoretical performance bounds of the explicit caching system. In our simulation, the total number of users is set to be 100, while the average number of users served by the BS is set to be 10. The available bandwidth *W* = 10 MHz and the maximum user delay tolerant is 1 s. The total number of files *F* is 1000. The files size follows uniform distribution in [1, 9]. Numerical results are obtained based on direct calculations from the derived equations, while empirical results are obtained by solving 100 independent (P3) optimization problems and taking numerical average over the 100 snapshots of optimal outcomes.

**Figure 3:**
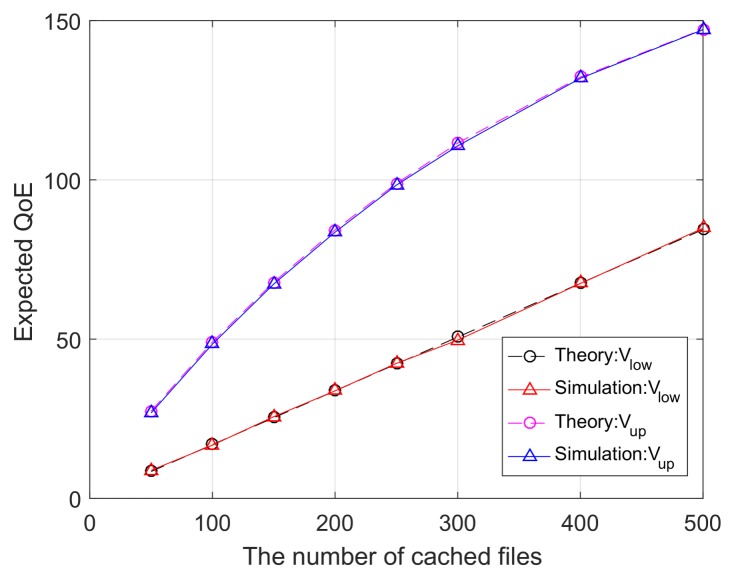
The expected QoE as a function of the number of cached files *M*.

Figure 3 shows the upper and lower bounds of the expected QoE as functions of the number of cached files *M*. It can be observed that the lower bound increases almost linearly with increasing *M*, while the upper bound increases with diminishing returns when *M* gets large. Moreover, the numerical (i.e., theoretical) and empirical (i.e., simulation) curves are shown to agree well. This serves as a validation to our theoretical derivations.

**Figure 4:**
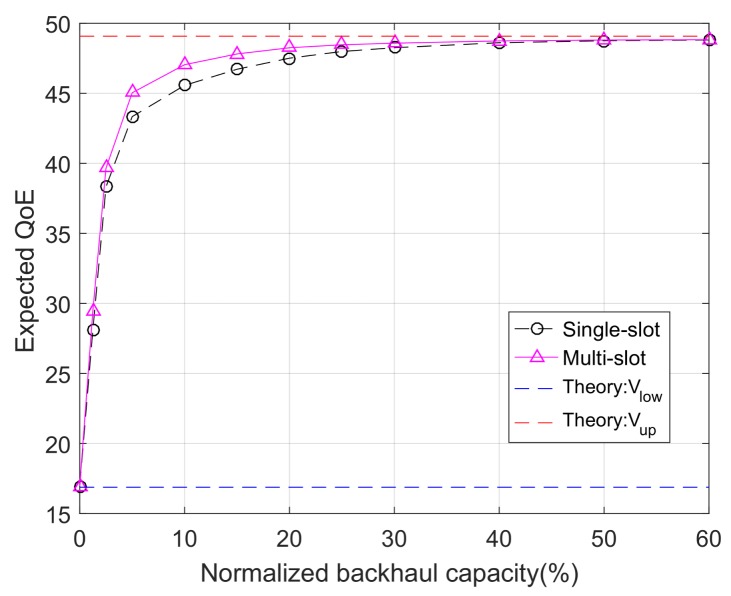
The expected QoE as a function of the normalized backhaul capacity (*M* = 100).

Figure 4 shows empirical results of the expected QoE as functions of the normalized backhaul capacity. Numerical/theoretical results on the upper and lower bounds of the QoE are also presented. It can be observed that, when the backhaul capacity gradually increases from zero to a large value, the empirical performance changes from the theoretical lower bound to the theoretical upper bound. This confirms our previous statement that the derived lower and upper bounds correspond to the two extreme cases of zero backhaul capacity and unlimited backhaul capacity, respectively.

## 6. Simulation Results and Discussions with Realistic Model

In this section, Monte Carlo simulations are performed to evaluate the performance of the explicit caching system with realistic modelling assumptions. The average number of users served by the BS is set to be 10 and the available bandwidth *W* is 10 MHz. The total number of users is set to be 100 and the maximum user delay tolerant is 1 s.

We consider a mixed content library containing two heterogeneous types of files: texts and videos. Different types of contents have different characteristics in terms of the file length distribution and user interest profile. Our simulations consider a library consisting of 500 text files and 500 video files. The lengths of text files and video files follow two log-normal distributions with mean values of 1 MB and 10 MB, respectively. The popularity of files follows a Zipf distribution with β=1 and $Z_{max}=80$. The users’ interest for a specific file follows a half-normal distribution with unit variance. We further assume that the QoE of video is 10 times that of texts, i.e., ξ=1 for texts and ξ=10 for videos. The SINR of users is assumed to be exponentially distributed with a mean value of 10 dB and truncated to have a minimum SINR of 3 dB.

Given the above settings, our simulation runs 100 snapshots. In each snapshot, the user interest profile, user association, and user channel state information, etc. are generated independently and randomly. The simulation results are averaged over the 100 snapshots.

### 6.1. Policies with Cross-Layer Information

This subsection aims to answer the following question: to what extent can cross-layer information contribute to improving the performance of explicit caching. Without loss of generality, our discussion is constrained to the single-slot optimization problem (i.e., N=1). We consider six different caching policies categorized into two types. The first type is policies that exploit physical or MAC layer information. We have three policies of this type, corresponding to the three cases discussed in Section 3. The second type is conventional caching policies that rely solely on upper layer information. Three representative policies of the second type are considered: the most popular content (MPC) policy [46], largest content diversity (LCD) policy [47], and largest popularity per unit content (PPU) policy, which caches contents based on the criteria of having large popularity per unit length. We note that the first type policies use optimal algorithms with exponential complexity, while the second type policies are greedy algorithms with polynomial complexity.

**Figure 5:**
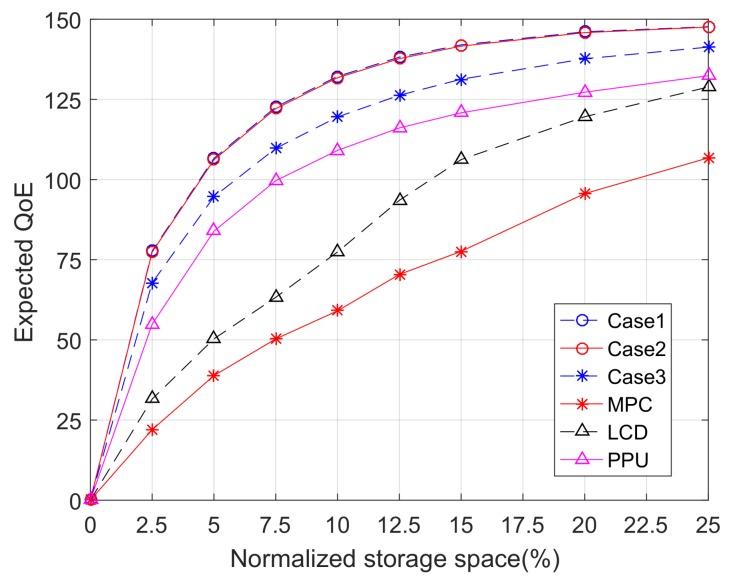
The expected QoE as a function of normalized storage space for different caching policies.

Figure 5 shows the expected user QoE as a function of the storage space when different caching policies are applied. The storage space is normalized by the total size of the file library. The backhaul capacity limit is set to be 50% of the storage space. Figure 5 shows that increasing the storage space can improve the QoE performance but has diminishing returns. Moreover, policies of the first type significantly outperform all policies of the second type. Among the three policies of the first type, Case 1 and Case 2 policies yield almost identical performance, while Case 3 gives a slightly worse performance. This suggests that the advantage of the first type policy mainly comes from exploiting the information of user association rather than the information of user SINR.

### 6.2. Impacts of Backhaul, Mobility, and Mobility Prediction

**Figure 6:**
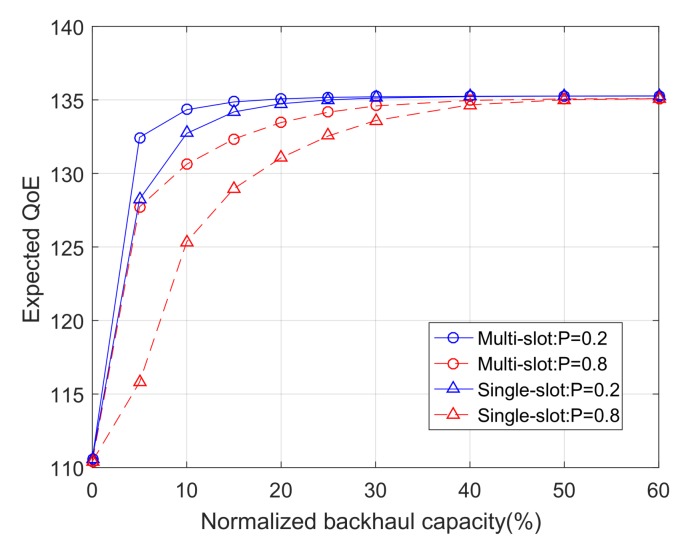
The expected QoE as a function of the normalized backhaul capacity, with varying user mobility and number of time slots.

Apart from cross-layer information, we are interested in the impacts of backhaul capacity, user mobility, and the number of jointly optimized time slots on the system performance. Without loss of generality, we choose the (near-optimal) Case 2 policy as our caching policy. Figure 6 shows the expected QoE per user as a function of the backhaul capacity. The backhaul capacity is normalized by the maximum backhaul capacity, which is set to be 500 MB. We consider two cases with N=1 (single-slot) and N=5 (multi-slot). Moreover, we consider two scenarios with high user mobility ($P_{a}=0.8$) and low user mobility ($P_{a}=0.2$).

Figure 6 reveals that high user mobility generally demands greater backhaul capacity, which is an intuitive result. However, it also shows that, when the backhaul capacity is very small or very large, the performances with high mobility and low mobility converge. This implies that mobility and backhaul capacity are relevant, but not limiting factors that will constrain the fundamental system performance. The system performance is fundamentally constrained by other factors such as bandwidth, content interest profile, and cache storage size, which can be easily seen from the utility function and storage constraint. Moreover, Figure 6 shows that the multi-slot algorithm outperforms the single-slot algorithm. We note that applying the multi-slot algorithm implies that users’ mobility (and hence user association) in N=5 time slots can be accurately predicted. The results of Figure 6 suggest that mobility prediction can be traded for backhaul capacity, but has no impact on the fundamental performance limit.

### 6.3. Impacts of User Density and File Popularity Distribution

**Figure 7:**
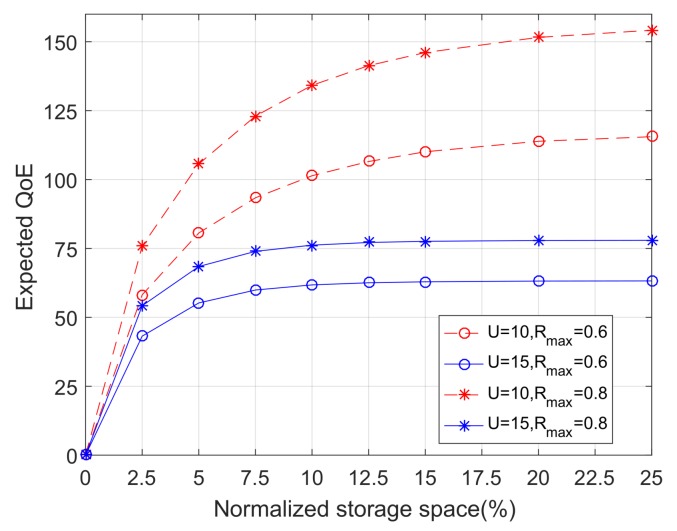
The expected QoE as a function of the normalized storage space, with varying values of the average number of users per BS and different distributions of the file popularity.

Figure 7 shows the expected QoE as a function of the normalized storage space with varying system parameters. First, Figure 7 illustrates the impact of the average number of users per BS on the QoE performance. It can be seen that, as the number of users *U* increases, the QoE performance degrades. This is because, when *U* increases, the downlink capacity allocated to each user reduces, which ultimately results in a reduction in the expected QoE. In addition, Figure 7 also shows how the QoE performance changes with different $R_{max}$($R_{max}=Z_{max}/K$), which represents the concentration of file popularity. It can be seen that, with increasing $R_{max}$, the performance of the system also increases due to a higher concentration of user interest in a given number of cached files.

### 6.4. Impacts of High SINR Approximation

**Figure 8:**
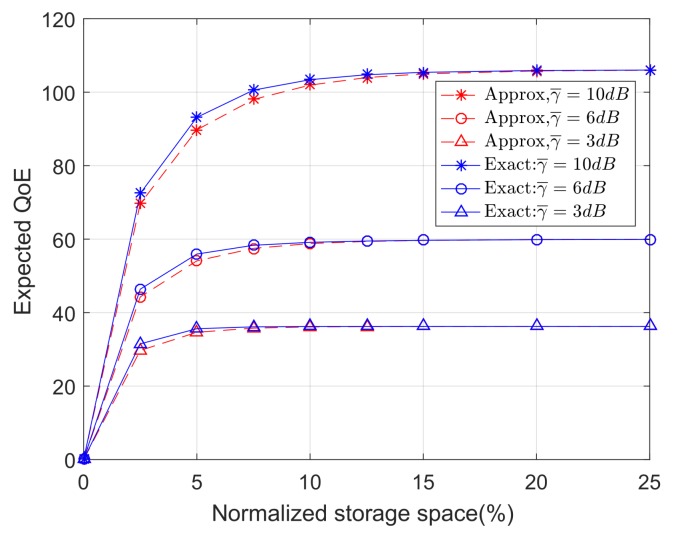
The expected QoE as a function of the normalized storage space with approximated and exact utility functions.

In Section 3.2, the high SINR approximation has been introduced to to compute the utility function (i.e., expected capacity) in closed-form. Alternatively, the exact value of the utility function can be obtained by the Monte Carlo method, which is computationally inefficient. To reveal the possible drawbacks of using the high SINR approximation, Figure 8 compares the system performance of two algorithms: the proposed algorithm that uses the high SINR approximation in its utility function, and the optimal algorithm that uses the Monte Carlo method (i.e., exact method) in its utility function. Results show that the high SINR approximation brings very small performance penalty even when the actual average SINR is small (e.g., 3 dB). These results suggest that the proposed algorithm with the high SINR approximation is robust for practical ranges of SINR.

### 6.5. Comparison between Algorithms

**Figure 9:**
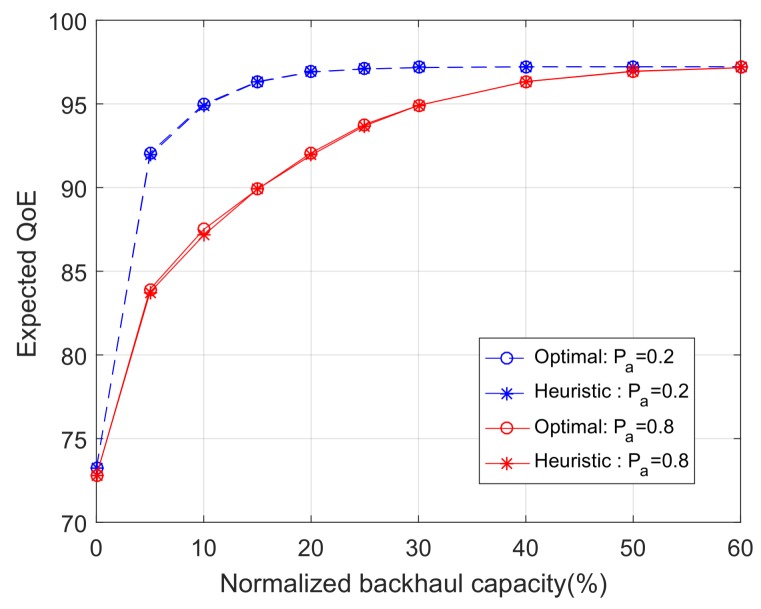
The expected QoE as a function of the normalized backhaul capacity in the single time slot optimization problem (*S* = 200 MB).

**Figure 10:**
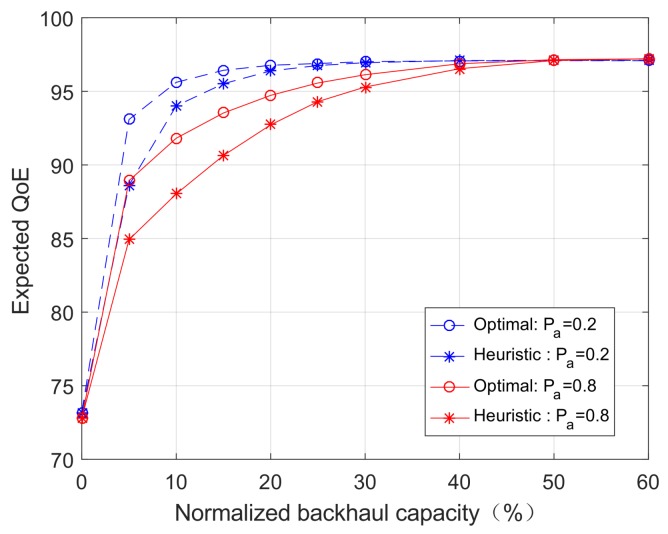
The expected QoE as a function of the normalized backhaul capacity in the multiple time slot optimization problem (*S* = 200 MB, *I* = 500).

In this subsection, we want to evaluate the performance gaps between the optimal algorithms and the heuristic algorithms proposed in Section 4. Figure 9 and Figure 10 compare the performance in single-slot and multi-slot scenarios, respectively. It can be seen in Figure 9 that, in the single-slot scenario, the proposed heuristic algorithm yield almost identical performance to the optimal algorithm. In the multi-slot scenario, Figure 10 shows that the performance gaps between heuristic and optimal algorithms are small in general, and diminishes in the extreme cases when the backhaul capacity approaches zero or becomes very large. These results suggest that the proposed heuristic algorithms are effective algorithms to be used in practice.

### 6.6. Discussions on Future Work

Finally, our work in this paper considers a simplified scenario of independent BS and independent backhaul. In practice, nearby BSs may cooperate to achieve better performance, and the backhaul links may be shared among multiple BSs. The cross-layer analytical framework established in this paper can be extended. Moreover, the results obtained in this paper can be used as heuristic or initial inputs for the more complicated cases of interdependent BSs. For example, in the shared backhaul scenario, we can formulate the problem of backhaul capacity allocation as a multi-level water-filling problem, using the QoE-backhaul relationship of each BS (as showed in Figure 9 and Figure 10 in this paper) as inputs to calculate the water levels. These promising extensions will be considered in our future work.

## 7. Conclusions

This paper proposes a novel BS caching paradigm called explicit caching. Optimal and near-optimal heuristic algorithms have been proposed to solve the content caching problem. Using user QoE as the ultimate performance metric, we have systematically investigated the theoretical and simulated performances of the explicit caching system. It has been revealed that the performance of an explicit caching system is fundamentally limited by the cache storage space, user interest profile, and available radio bandwidth, while increasing backhaul capacity, exploiting cross-layer information, and having user mobility prediction can only contribute to better approaching the fundamental performance bounds. We conclude that the explicit caching system is a novel and promising edge computing application for personalized content dissemination over mobile edge networks.

## Figures and Tables

**Table 1:** Major system parameters and their physical meanings.

System Symbols	Physical Meaning of Symbols	System Symbols	Physical Meaning of Symbols
K	The set of users that may be served by the BS over a long term	*K*	The size of K
U	The set of connected users to the BS	*U*	The size of U
F	The large content pool	*F*	The size of F
$D_{k,f}$	The time required for the *k*-th user to download the *f*-th file	$Q_{k,f}$	The QoE for the *k*-th user to download and view the *f*-th content
$I_{k,f}$	The interest of the *k*-th user on the *f*-th file	$Z_{k,f}$	Delivery evaluation for the *k*-th user to download the *f*-th content
$L_{f}$	The file length	$C_{k}$	Instantaneous downlink capacity of the *k*-th user
$T_{s}$	Duration of one time slot	$T_{0}$	Time threshold of effective distribution
*W*	Radio bandwidth	$P_{t}$	Transmit power per user
$d_{k}$	Distance between BS and the *k*-th user	α	The path-loss exponent
$g_{m}$	Fast fading coefficient	$\sigma^{2}$	The power spectrum density of sum noise and interference in the cell
$\gamma_{k}$	The *k*-th User’s SINR averaged over small scale fading	ξ	Scaling constant representing the QoE of an effective distribution
*S*	Cache storage space of the BS	δ	Backhaul capacity of the BS
